# Metabolomic Analysis Reveals Unique Biochemical Signatures Associated with Protection from Radiation Induced Lung Injury by Lack of *cd47* Receptor Gene Expression

**DOI:** 10.3390/metabo9100218

**Published:** 2019-10-08

**Authors:** Elizabeth R. Stirling, Katherine L. Cook, David D. Roberts, David R. Soto-Pantoja

**Affiliations:** 1 Department of Cancer Biology, Wake Forest School of Medicine Comprehensive Cancer Center, Winston-Salem, NC 27101, USA; estirlin@wakehealth.edu (E.R.S.); klcook@wakehealth.edu (K.L.C.); 2 Wake Forest School of Medicine Comprehensive Cancer Center, Winston-Salem, NC 27101, USA; 3 Department of Surgery, Wake Forest School of Medicine Comprehensive Cancer Center, Winston-Salem, NC 27101, USA; 4 Laboratory of Pathology, Center for Cancer Research, National Cancer Institute, National Institutes of Health, Bethesda, MD 20892, USA; droberts@mail.nih.gov; 5 Department of Radiation Oncology, Wake Forest School of Medicine Comprehensive Cancer Center, Winston-Salem, NC 27101, USA

**Keywords:** ionizing radiation, radiation-induced lung injury, CD47, thrombospondin-1, redox, amino acid, fatty acid, lipid, xenobiotics

## Abstract

The goal of this study was to interrogate biochemical profiles manifested in mouse lung tissue originating from wild type (WT) and *cd47* null mice with the aim of revealing the in vivo role of CD47 in the metabolic response to ionizing radiation, especially changes related to the known association of CD47 deficiency with increased tissue viability and survival. For this objective, we performed global metabolomic analysis in mouse lung tissue collected from (C57Bl/6 background) WT and *cd47* null mice with and without exposure to 7.6 Gy whole body radiation. Principal component analysis and hierarchical clustering revealed a consistent separation between genotypes following radiation exposure. Random forest analysis also revealed a unique biochemical signature in WT and *cd47* null mice following treatment. Our data show that *cd47* null irradiated lung tissue activates a unique set of metabolic pathways that facilitate the handling of reactive oxygen species, lipid metabolism, nucleotide metabolism and nutrient metabolites which may be regulated by microbial processing. Given that *cd47* has pleiotropic effects on responses to ionizing radiation, we not only propose this receptor as a therapeutic target but postulate that the biomarkers regulated in this study associated with radioprotection are potential mitigators of radiation-associated pathologies, including the onset of pulmonary disease.

## 1. Introduction

Lack of selective radioprotectants for normal tissue limits the therapeutic doses that can be delivered to treat cancers and limits the therapeutic strategies in place to treat the onset of Acute Radiation Syndrome due to accidental or intentional exposure to ionizing radiation [1,2]. Furthermore, pulmonary tissue is exquisitely sensitive to radiation damage and is considered treatment-limiting in patients exposed to total-body irradiation (TBI) in preparation for hematopoietic stem cell transplantation [3]. Because of its sensitivity to radiation, lung tissue is vulnerable to early and late effects of radiation that can impact quality of life, survival and future risk to pulmonary pathologies [4,5]. Thus, finding biomarkers and strategies to modulate tissue responses to stress is an important therapeutic objective to treat the oxidative and genotoxic stresses caused by exposure to ionizing radiation.

Cluster of differentiation 47 (CD47, encoded by *cd47* in mouse) is a signaling receptor for thrombospondin-1 (TSP1) and an attractive cancer therapeutic target as blocking CD47 signaling protects normal tissue while sensitizing tumors to ionizing radiation [6,7,8]. Moreover, CD47 is also a target for a potential defense countermeasure drug as decreasing CD47 expression increases survival of mice exposed to lethal doses of whole body ionizing radiation [9]. CD47 is a widely expressed receptor that controls cell fate via two major functions: (1) interaction with signal regulatory protein alpha (SIRPα) on phagocytic cells, which results in inhibition of phagocytic activity and (2) binding to TSP1. CD47 transduces signals that alter cellular calcium, cyclic nucleotide, integrin, growth factor signaling and controls cell viability and resistances to stress [10,11]. This latter function is fundamental to understanding why targeting CD47 could provide therapeutic benefits to treat radiation-induced pathologies. In previous studies we demonstrated that the radioprotective effect of CD47 on cells and tissues, including lungs, was mediated through the activation of protective autophagy [9,12]. Furthermore, in vitro studies with WT and CD47-deficient T lymphocyte cell lines demonstrated that the absence of this receptor globally impacted metabolic pathways to overcome stress associated with ionizing radiation treatment [13].

In this new report we present a global overview of the in vivo modulation of metabolism by the receptor CD47 in response to whole body irradiation at 24 h. This time point was selected due to government guidelines suggesting that medical countermeasure delivery would be able to be started 24h post-exposure [14] Furthermore, one of the aims of radiation biodosimetry efforts is to elucidate early biomarkers to streamline triage of victims after a radiation incident [1]. The present study was conducted using Liquid chromatography/Mass spectrometry (LC-MS) and a Gas chromatography/Mass spectrometry (GC-MS) analysis platform with the objective of identifying metabolic perturbations in WT and *cd47* null lung tissue following exposure to ionizing radiation. The use of these platforms allows an in-depth metabolite discovery to understand physiological responses to ionizing radiation exposure [1] and allows us to gain a profound understanding of the metabolic regulation associated with the radioprotection of cells and soft tissues we have previously observed with the blockade or deficiency of the receptor CD47. 

Our data show that *cd47* null irradiated lung tissue activates a unique set of metabolic pathways that facilitate the handling of reactive oxygen species (ROS), lipid metabolism, nucleotide metabolism and nutrient metabolites which may be regulated by microbial processing. Given that CD47 has pleiotropic effects on responses to ionizing radiation, we not only propose this receptor as a sole target but postulate that the biomarkers regulated in this study associated with radioprotection are considered potential mitigators of radiation-associated pathologies including the onset of pulmonary disease.

## 2. Results

### 2.1. Unique Biochemical Signatures in the Absence of CD47 after Ionizing Radiation Treatment

**Figure 1:**
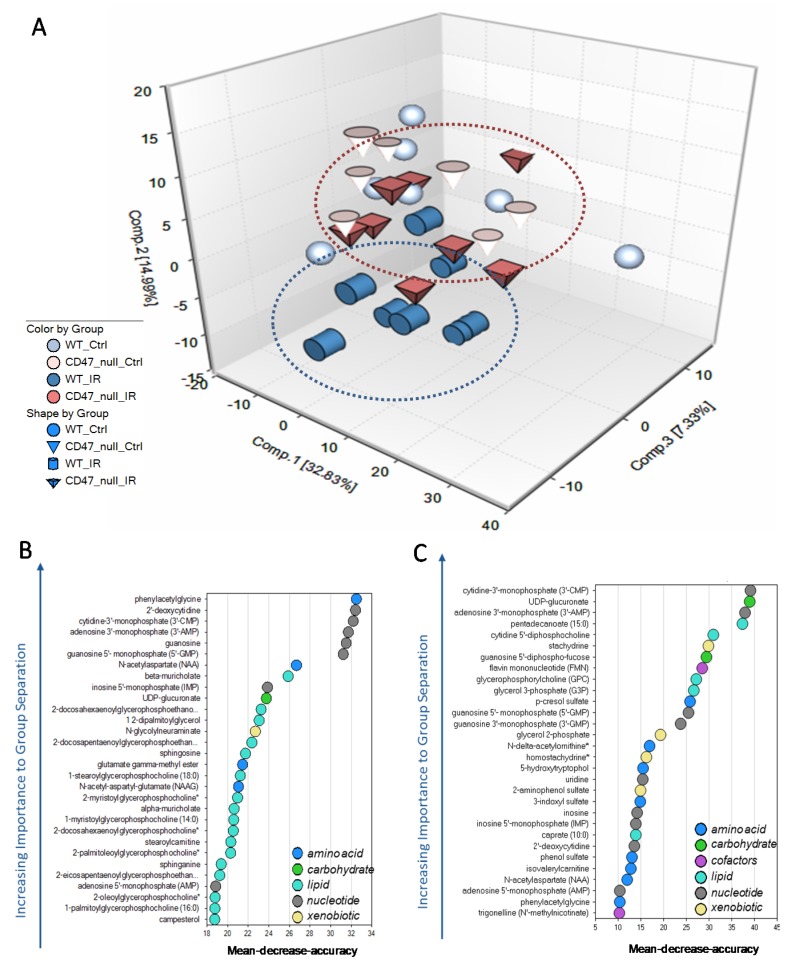
WT and *cd47* null mice demonstrate different biochemical signatures after exposure to ionizing radiation. WT and *cd47* null mice were left untreated or were exposed to whole body irradiation (7.6 Gy). After 24 h, lungs were harvested and processed for metabolomic analysis. (**A**) Principal component analysis of lung tissues of WT and *cd47* null mice with or without exposure to whole body irradiation. Random forest analysis of (**B**) WT irradiated or (**C**) *cd47* null lung irradiated tissues. n = 7–8/group.

The present study identified over 300 compounds of known identity in lung tissue. Following median scaling, imputation of missing values, if any, with the minimum observed value for each compound and log transformation median scaled data, analysis of variance (ANOVA) contrasts were used to identify biochemicals that differed significantly between experimental groups. Principal component analysis revealed a consistent separation between WT and *cd47* null tissues following irradiation exposure which was supported by random forest analysis (Figure 1A, Supplemental Figure S1A). Furthermore, random forest analysis also indicated a unique biochemical signature in WT and *cd47* null mice after treatment with a predictive accuracy of 87% and 100%, respectively (Figure 1B,C). Notably, neither principal component nor random forest analysis demonstrated a separation between genotypes without treatment (Figure 1A and Supplemental Figure S1B), suggesting that *cd47* primarily regulates a metabolic response to damage after stress such as ionizing radiation.

### 2.2. *CD47* Modulates Antioxidant Response after Ionizing Radiation

**Figure 2:**
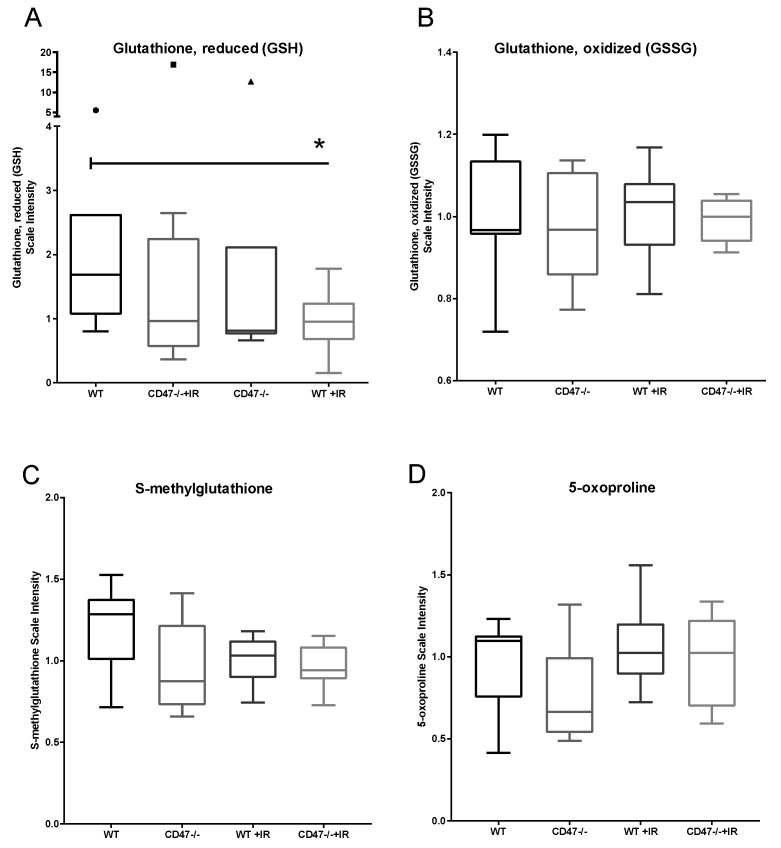
Regulation of glutathione metabolism. (**A**) Reduced (GSH) and (**B**) oxidized (GSSG) glutathione were measured along with metabolite precursors (**C**) (S-methylglutathione) and (**D**) 5-oxoproline. Symbols denote outliers. (* *p* < 0.05, *n* = 7–8).

One of the most well characterized pathways in studying tissue response to ionizing radiation is the glutathione pathway. Reduced glutathione (GSH) is a major antioxidant and is associated with protective response to ionizing radiation [15]. On the other hand, oxidized glutathione (GSSG) is associated with damage and oxidative stress and thus found at lower levels in normal conditions. Furthermore, the ratio of these two metabolites can serve as a measure of oxidative damage with a ratio favoring GSH associated with radioprotection. As expected, our metabolomics data showed that GSH was more abundant than GSSG in WT and *cd47* null animals with no significant differences between genotypes (Figure 2A,B). However, after radiation there was a significant 66% drop in GSH levels in WT animals, and the ratio of GSH to GSSG was higher in *cd47* null irradiated animals (Figure 1A and Table 1), which is consistent with previous literature examining these metabolites during radiation exposure [13]. A similar trend was also observed in S-methyl glutathione, another derivative of GSH (Figure 2C). On the other hand, *cd47* null irradiated tissues showed slight elevated levels of GSH when compared to WT and *cd47* null control animals. The levels of the precursor of glutathione, 5-oxoproline, remained similar among genotypes regardless of treatment, suggesting that this regulation is not due to elevated levels of the precursor metabolite in *cd47* null mice (Figure 2D). Consistent with our previous data in irradiated CD47-deficient T cells, the ratio of GSH to GSSH was higher in irradiated *cd47* null tissues when compared to irradiated WT lung, confirming an enhanced antioxidant response to oxidative stress that occurs acutely in CD47-deficient cells after exposure to ionizing radiation. Another pathway implicated in the regulation of oxidative stress after ionizing radiation was the metabolism of heme. Heme is a cofactor bound to hemoglobin and can be converted to biliverdin by heme oxygenase to biliverdin [16,17]. Our data showed that heme is significantly elevated in WT mice after radiation; however no significant differences were observed in biliverdin (Supplemental Figure S3A,B). Consistent with other studies, this suggests that heme metabolism may be a marker of radiation exposure in lungs [17], but together with the data presented above, suggests that CD47 regulates an antioxidant response through the regulation of glutathione metabolism.

### 2.3. *CD47* Regulates Lipid Metabolism as a Response to Ionizing Radiation

**Figure 3:**
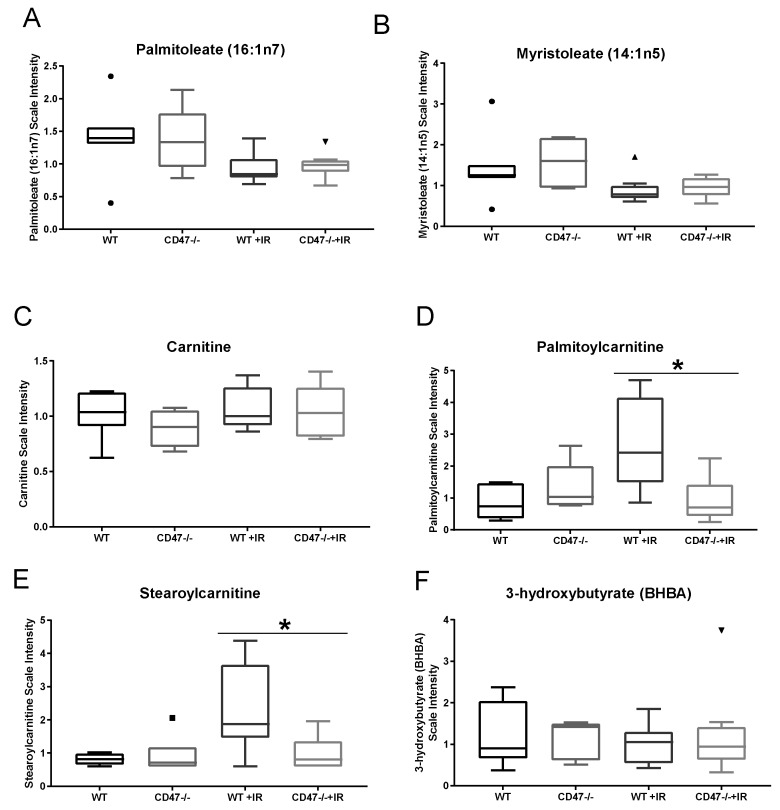
Regulation of lipid metabolism in lungs as a response to ionizing radiation. Lipid metabolites (**A**) Palmitoleate, (**B**) Myriostoleate, (**C**) Carnitine, (**D**) Palmitocarnitine, (**E**) Stearoylcarninitne and (**F**) BHBA of lung tissues were measured 24 h after exposure to whole body irradiation. Symbols denote outliers. (* *p* < 0.05, *n* = 7–8).

We have previously reported that glycolysis and citric acid intermediates participate in response to stress in both WT and *cd47* null T cells and lung tissue with an observed reduction in citrate levels in WT irradiated lungs when compared to *cd47* null [13]. While other metabolites downstream of citrate in the TCA cycle may be regulated, our statistical analysis suggested that metabolic demands after ionizing radiation may be met by activation of fatty acid synthesis. Irradiated WT and *cd47* null mice exhibited a modest decrease in long chain fatty acid metabolites such as palmitoleate and myristoleate, reflecting possible changes in lipid synthesis and oxidation (Figure 3A,B). While carnitine levels were not different between WT and *cd47* null irradiated tissues (Figure 3C), the carnitine conjugated lipids palmitoylcarnitine and stearoylcarnitine significantly accumulated in irradiated WT lungs, but not in irradiated *cd47* null lungs (Figure 3D,E). The accumulation of these carnitine conjugated lipids may be indicative of a defect in lipid import into the mitochondria for subsequent oxidation. Consistent with this observation and previous data from irradiated CD47-deficient T cells (Supplemental Figure S2A), 3-hydroxybutyrate levels, a metabolite marker of β-oxidation, was reduced in WT but not irradiated *cd47* null tissues (Figure 3F) [13]. This suggests that fatty acid synthesis and oxidation remain intact in *cd47* null lungs, providing an additional potential energy source for tissue repair and survival.

### 2.4. Differential Regulation of Cell Membrane Phospholipids after Ionizing Radiation

**Figure 4:**
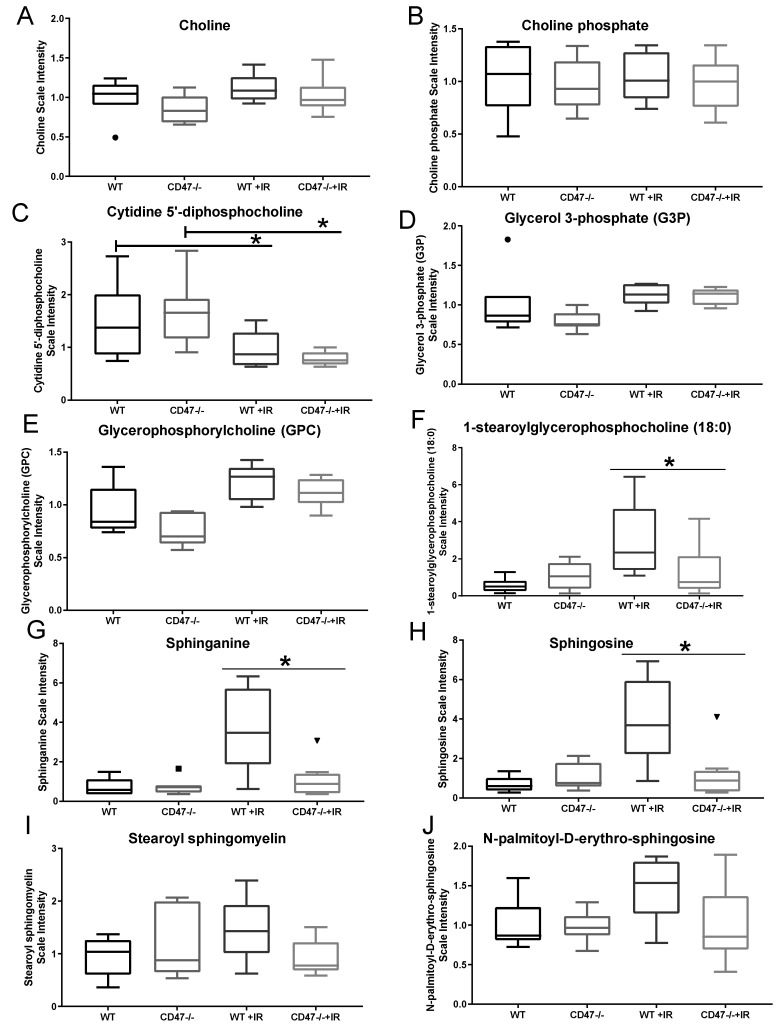
Regulation of cell membrane lipid metabolites after exposure to ionizing radiation. (**A**) Choline, (**B**) Choline phosphate, (**C**) Cytidine 5′-disphosphocholine, (**D**) Glycerol 3-phosphate, (**E**) Glycerophosphorycholine, (**F**) 1-stearolglycerophosphocoline, (**G**) Sphingannine, (**H**) Sphingosine, (**I**) Stearoyl sphiongomyelin and (**J**) N-palmitoyl-D-erythro-sphingosines were measured in lungs of mice exposed to ionizing radiation. Symbols denote outliers. (* *p* < 0.05, *n* = 7–8).

Another aspect of lipid metabolism regulated in our study was the synthesis of membrane phospholipids, which are essential in the maintenance of cellular structure as a response to ionizing radiation [18]. The synthesis of phospholipids is achieved by the catalysis of choline and ethanolamine (known as the Kennedy Pathway or CDP-choline pathway) to produce phosphocholine and phosphoethanolamine, respectively, to the eventual production of glycerophosphocholine and glycerophosphoethanolamine [19]. Choline levels were moderately elevated after radiation in both genotypes, while downstream metabolite choline phosphate (Phosphocholine) remained equal among groups (Figure 4A,B). On the other hand, downstream phospholipid precursor cytidine 5′-diphosphocholine was significantly reduced in irradiated tissues when compared to the control counterparts (Figure 4C), suggesting that this metabolite may be a marker of radiation injury and implying a compensatory mechanism to synthetize membranes after insult. This was further confirmed by the increase in glycerol 3-phosphate and glycerophosphocholine metabolite levels in both irradiated groups, thus supporting a lipid hydrolysis mechanism occurring as a response to radiation in the choline arm of the Kennedy pathway (Figure 4D,E). This was further supported as we did not observe changes in ethanolamine and phosphoethanolamine among groups with or without radiation (Supplemental Figure S3C,D). However, stark differences were observed in lysolipids such as 1-steraoryl gycerophosphocholine accumulation in WT irradiated tissues when compared to *cd47* null irradiated tissues (Figure 4F), thus suggesting that irradiated WT cell membranes have elevated turnover due to higher damage when compared to *cd47* null cells. Aside from changes in phospholipid metabolism, the sphingolipid metabolites sphinganine, sphingosine, stearoyl sphingomyelin, and N-palmitoyl-D-erythro-sphingosine accumulated in irradiated WT lungs (Figure 4G–J). In contrast, this trend was not significantly observed in irradiated *cd47* null mice and may suggest a difference in tissue injury between genotypes as sphingolipids are critical regulators of cell growth and survival (Table 2).

### 2.5. Absence of CD47 Preserves Nucleotide Metabolism after Ionizing Radiation

**Figure 5:**
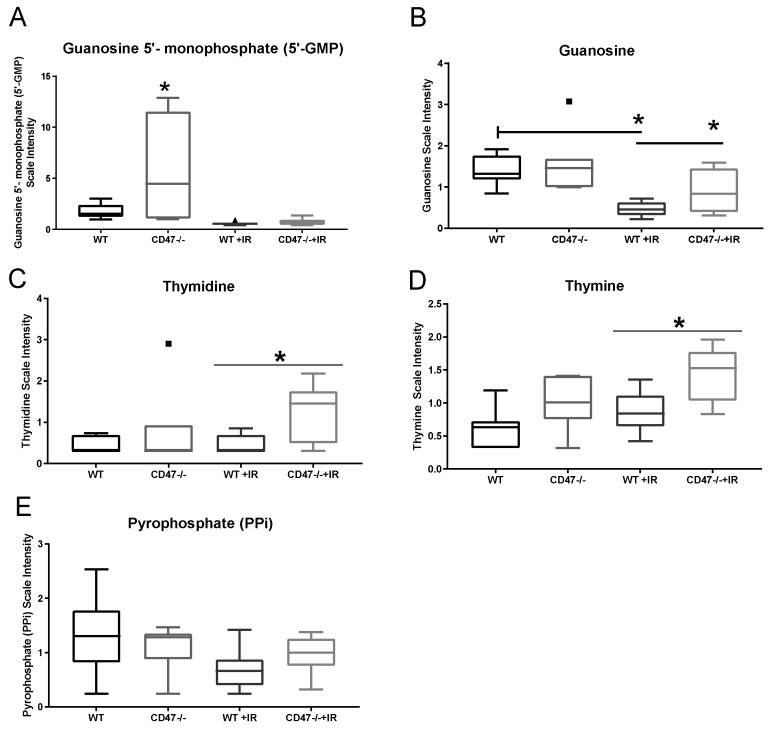
Regulation of nucleotide metabolism. Purines metabolism metabolites (**A**) Guanosine 5′-monophosphate (**B**) Guanosine and Pyrimidine metabolism metabolites (**C**) Thymidine, (**D**) Thymine was measured in lungs of mice exposed to IR. (**E**) Pyrophosphate metabolite levels were measured as a surrogate of nucleotide metabolism activity. Symbols denote outliers. (* *p* < 0.05, *n* = 7–8).

Nucleotide biosynthesis is essential for mechanisms of DNA repair triggered by strand breakage caused by ionizing radiation. As expected, key intermediaries of purine metabolism were depleted in both groups after ionizing radiation (Table 3). However, some key differences were observed between genotypes. As observed in Figure 5A, levels of 5-guanosine monophosphate (GMP) were elevated in *cd47* null lungs when compared to WT, which may explain the slight elevated levels of this metabolite in *cd47* null radiated tissue when compared to WT irradiated. However, downstream metabolism of GMP by nucleotidases generates guanosine, which was found to be significantly increased in the *cd47* null irradiated tissues when compared to WT irradiated (Figure 5B). This suggests that the elevated levels of GMP preceding radiation in *cd47* null tissues may provide an advantage to the synthesis of purines by generating guanosine. In terms of pyrimidine metabolism, we observed that most of the metabolites detected remained mostly stable even with ionizing radiation (Table 3). Still, significantly elevated levels of thymine were observed in *cd47* null tissues when compared to WT, and a further elevation of thymine was observed in *cd47* null tissues after irradiation (Figure 5D). This would indicate that thymine may accumulate in *cd47* null mice after irradiation. However, there were significantly increased levels of thymidine (Figure 5C) in *cd47* irradiated mice, which is converted to thymine by the action of nucleosidases, suggesting an enhanced pathway for nucleoside metabolism in the *cd47* null condition. Furthermore, pyrophosphate is released when nucleotides are incorporated into DNA, which is linked to radiation-induced DNA damage [13]. Our studies showed a reduction in this metabolite in WT mice after irradiation, whereas there was no significant change in the *cd47* null tissues after irradiation (Figure 5E). The drop in pyrophosphate levels in WT mice suggests a deficiency in the capacity for DNA repair and synthesis after ionizing radiation treatment. This together with the aforementioned changes in nucleoside metabolites suggest that deficiency of *cd47* plays a key role in the regulation of nucleotide metabolism, which would allow better recovery of tissue after radiation exposure.

### 2.6. Differential Regulation of Nutrient Processing after Ionizing Radiation

**Figure 6:**
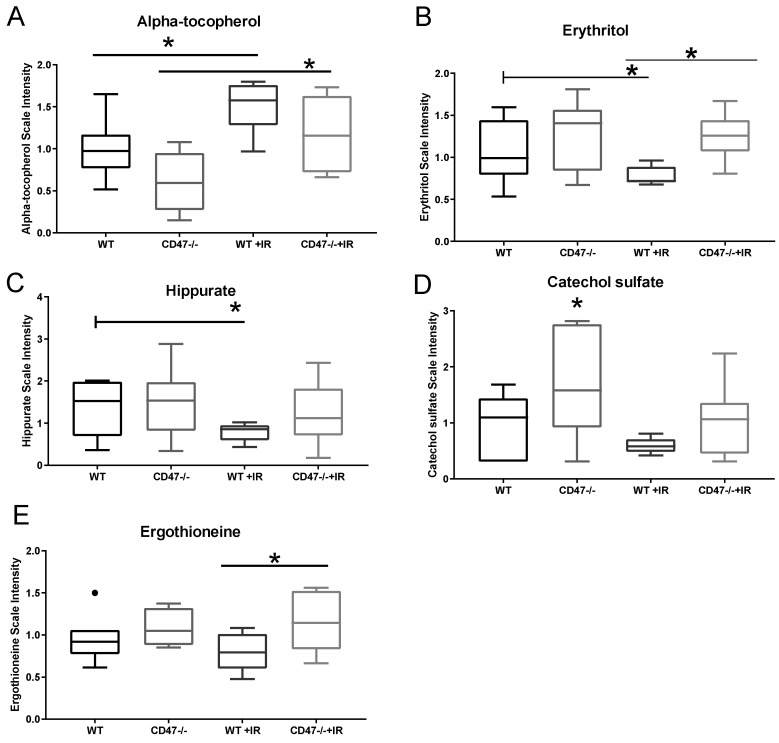
Regulation of nutrient and xenobiotic metabolite processing after exposure to ionizing radiation. (**A**) alpha-tocopherol, (**B**) Erythritol, (**C**) Hippurate, (**D**) Catechol sulfate and (**E**) Ergothioneine metabolites were measured in lungs of mice with or without whole body irradiation. Symbols denote outliers. (* *p* < 0.05, *n* = 7–8).

Exposure to whole body irradiation causes changes in nutrient absorption and metabolism, thus co-factors and vitamins have been studied as potential intervention as countermeasure drugs. The heavily studied metabolite alpha-tocopherol (vitamin E) [2] was significantly elevated in WT and *cd47* null mice exposed to ionizing radiation when compared to their respective controls (Figure 6A). Given the antioxidant properties of this metabolite, it is possible that this metabolite is increased after ionizing radiation to mitigate damage. Another dietary component that was regulated in our study was erythritol, which can occur endogenously or come from food sources [20] (Figure 6B). Our data showed that WT irradiated mice had lower levels or this metabolite when compared to control; however, levels of erythritol remained stable in the *cd47* null mice after radiation, thus suggesting differences in nutrient metabolism between genotypes that may participate in responses to ionizing radiation. Supporting this observation, metabolites of benzoate metabolism were differentially regulated between genotypes after exposure to ionizing radiation. Sodium benzoate is a widely used food preservative that is metabolized to hippurate in the mitochondrial matrix [21]. Consistent with a *cd47*-dependent depletion of hippurate in irradiated T cells (Supplemental Figure S2B), our data showed that this metabolite was depleted after exposure to ionizing radiation in WT mice but was preserved in *cd47* null mice (Figure 6C). Since mitochondrial processing of hippurate can impact ATP and sugar metabolism, these results suggest that nutrient handling in the *cd47* null mice may contribute to the previously observed protection of lung tissue and survival of these mice to ionizing radiation. Furthermore, microbial degradation of benzoate can also lead to the production of catechol substrates. Our data showed elevated catechol sulfate levels in *cd47* null mice lungs when compared to WT and remained elevated in *cd47* null mice when compared to WT irradiated mice (Figure 6D). Consistent differences were found in *cd47* null T cells exposed to ionizing radiation when compared to WT (Supplemental Figure S2C). This not only indicates differential regulation of food metabolites between genotypes but also shows differential microbial processing of metabolites as a consequence of ionizing radiation. Another aspect that suggests differential microbial processing is the observed regulation of xenobiotics. Ergothioneine, a metabolite produced by Actinomycetales and cyanobacteria, was elevated in *cd47* null mice when compared to WT before and after ionizing radiation (Figure 6E). Ergothioneine has been demonstrated to have antioxidant properties [22], thus suggesting that the elevated presence of this metabolite in the *cd47* null mice may provide an advantage in the protective response to ionizing radiation. Therefore, our data suggest that differential metabolism of nutrients either by mitochondrial or microbial processing may play a role in the observed radioprotection of *cd47* null mice. 

## 3. Discussion

We had previously examined the contribution of CD47 deficiency to radiation resistance between WT and CD47 deficient Jurkat T lymphocytes cell line and observed that CD47 deficient cells and lung tissues were protected from increasing doses of ionizing radiation by the activation of autophagy [12]. Autophagy is a highly evolutionary cellular process that is heavily regulated in part by nutrient availability and metabolism [23]; thus, we performed untargeted metabolomics and discovered the CD47 deficient cells globally upregulated anabolic metabolism as a survival response to radiation treatment. We observed in these cells profound changes in metabolic pathways controlling oxidative stress, DNA repair and energy metabolism [13]. The latter was validated in lung tissue irradiated at 24 h by examining levels of glucose, glucose-6-phosphate, fructose-6-phosphate, ribose-5-phosphate, sedoheptulose-7-phosphate, citrate and malate [13]. Moreover, we have previously demonstrated that CD47 blockade increase survival of mice exposed to total body irradiation, supporting our evidence of protection in cells and soft tissues [9]. Therefore, our aim in this study was to provide a comprehensive global survey of metabolites regulated by ionizing radiation in vivo in WT and *cd47* null mice, providing potential biomarkers of damage by ionizing radiation as well as metabolites associated with radioprotection, both important in the cancer therapy setting as well as in defense countermeasures research.

One of the early effects of ionizing radiation exposure is the accumulation of ROS, which mediates tissue damage and can lead to long-term tissue injury. Our study shows that reduced glutathione is more abundant in *cd47* null tissues and is maintained after exposure to ionizing radiation. This is also observed when considering ratios of reduced and oxidized glutathione, which remain higher in the *cd47* null irradiated mice vs. WT irradiated. Glutathione is formed by enzymatic action of glutamate–cysteine ligase, causing the addition of cysteine to glutamate to form the precursor γ-l-Glutamyl-l-cysteine [24]. Levels of glutamate and cysteine remained similar among groups (Supplemental Figure S3E,F), suggesting that the differences in GSH synthesis are not due to regulation of glutamate-cysteine ligase. However, levels of glycine, which is needed by glutathione synthetase, were reduced suggesting increased utilization of this metabolite in *cd47* null mice by upregulation of this enzyme. Furthermore, ligation of CD47 by TSP1 increases ischemic injury [25]. The TSP1/CD47 signaling axis modulates ROS production by regulating calcium-mediated eNOS activation, NO signaling, and NOX activation in several animal models [26,27,28]. In human vascular smooth muscle cells, the TSP1/CD47 complex activates phospholipase C and protein kinase C to phosphorylate the NOX organizer subunit p47^*phox*^ and increases Nox1-derived O_2_^•−^ generation in an ischemia-reperfusion model of cell injury. In endothelial cells, TSP1/CD47 signaling results superoxide generation through NOX-1 and eNOS decoupling, causing impaired vasoconstriction and poor recovery from ischemic injury [29]. Thus, these pathways may be responsible for lung radiation injury and prevented by absence of CD47 by the regulation of glutathione metabolism. Moreover, the antioxidant capacity observed in *cd47* null mice may also be supported by other pathways other than glutathione metabolism. We observed that the metabolite ergothioneine was upregulated in *cd47* null irradiated mice over WT. Egothioneine is thought to be a naturally occurring amino acid with a 2-mercaptoimidazole moiety [22]. This metabolite can be consumed from plant and animal sources but is only produced by non-yeast fungi, Actinomycetales and cyanobacteria [22]. Interestingly, in certain bacterial strains ergothioneine biosynthesis follows the condensation of glutamate and cysteine to form gamma-glutamate-cysteine, which is similar to glutathione biosynthesis [22]. This metabolite is thought to be a scavenger of ROS and has cytoprotective properties. Thus, the regulation of this metabolite is also consistent with our previous observation that blockade or absence of CD47 is protective against death from ionizing radiation exposure. This also indicates potential genotype differences in microbial processing of metabolites that may confer an advantage to the *cd47* null mice. Whether absence of *cd47* regulates specific microbiota species is not known, future studies should consider this aspect in understanding the mechanism of action of CD47.

We have previously demonstrated that blockade of *cd47* preserves mitochondria function after exposure of cells to ionizing radiation and preserves TCA intermediates associated with cytoprotection [9,11,12,13]. While the regulation of some of these intermediates was consistent in vivo, our random forest analysis indicates a strong regulation of lipid and fatty acid metabolism that can serve as potential energy sources to maintain biosynthetic pathways after exposure to irradiation. We observed that carnitine conjugated lipids accumulated in the WT irradiated tissues, which can suggest a defect in lipid import to the mitochondria for β-oxidation. β-oxidation leads to the production of acetyl-CoA that can be shuttled to the TCA cycle. Therefore, radiation treatment of WT tissues may inhibit carnitine palmitoyltransferase (CPT1) activity and expression required for the transport of long chain fatty acids into the mitochondria for oxidation [30]. On the other hand, deficiency of CD47 stimulated CPT1b in brown adipose tissue resulted in increased lipid utilization [31]. Furthermore, TSP1 can inhibit myristate-stimulated cGMP synthesis by engaging its receptor *cd47* [32]. Thus, the regulation of fatty acid synthesis and lipid metabolism may not only be due to inhibition by ionizing radiation but by the stimulation of a CD47 regulated pathway. 

The synthesis of phospholipids is essential for the maintenance of cell membrane integrity during stress [18]. Our data showed that the choline arm of the phospholipid synthesis pathway was affected by ionizing radiation when compared to the ethanolamine arm of this pathway, as most of the metabolites associated with this arm of phospholipid synthesis remained consistent between phenotypes regardless of radiation exposure. The drop in cytidine 5′-diphosphocholine and elevated levels of glycerol-3-phosphate in both WT and *cd47* null mice suggest a compensatory reaction to overcome damage to radiation and suggest a possible reliable marker for radiation response. Related to lipid metabolism, we observed that sphingolipid metabolites accumulated in irradiated WT lungs whereas these remained consistent between *cd47* null mice with or without radiation exposure when compared to untreated WT mice. The accumulation of ceramides such as N-palmitoyl-D-erythro-sphingosine is often associated with toxicity [33], and accumulation of ceramides can lead to apoptosis [34]. Indeed, sphingosine kinase is increased after lung irradiation, shifting the ratio of ceramides to sphingosines and leading to alterations in lung barrier integrity [34]. This shift caused by sphingosine kinase is also reported to cause long-term complications of radiation-induced lung pathologies such as fibrosis, and thus the inhibition of de novo synthesis of sphingolipids has been considered for the treatment of radiation lung injury complications such as pneumonitis [35]. Since the levels of sphingosines remained low in irradiated *cd47* null mice compared to irradiated WT, sphingosine kinase activity may be lowered by CD47 blockade, and thus future studies could be developed to determine if this is a feasible strategy to treat lung pathologies associated with radiation exposure. 

An important factor to overcome radiation injury is the ability to maintain intact DNA repair pathways. Our data show that *cd47* null mice have elevated levels of guanosine and thymidine, which can provide an advantage in repair by activation of nucleotide metabolism. While CD47 has not been directly linked to DNA repair pathways, its ligand TSP1 is reported to induce γ-H2AX in retinoblastoma [36]. Therefore, it is possible that upregulation of TSP1 as a consequence of radiation can regulate DNA integrity via *cd47*.

Dietary supplementation is of interest for the discovery of defense countermeasure drugs to ameliorate the effects of Acute Radiation Syndrome. This is evidenced by several studies examining the role of α-tocopherol (Vitamin E) derivatives in preserving the survival of animals exposed to ionizing radiation [37]. Our metabolomics data showed that α-tocopherol was elevated after radiation in both genotypes. Since α-tocopherol derivatives are known to be radioprotective [38], we hypothesize that processing of this metabolite may occur as a response to overcome the lethal effects of radiation exposure in both our animal genotypes. Another pathway regulated by ionizing radiation related to food consumption was benzoate metabolism. Benzoate is a common food additive that can be conjugated in the mitochondrial matrix with glycine to form hippurate [21]. Our data showed that hippurate levels dropped significantly as a consequence of radiation but were maintained in *cd47* null radiated lungs. Similar preservation of hippurate in irradiated CD47-deficient T cells indicates that this effect of CD47 on benzoate metabolism is cell autonomous [13]. Hippurate biosynthesis occurs in the mitochondrial matrix [39], and we know that CD47 blockade increases mitochondrial biogenesis [40] and preserves mitochondrial function after radiation [13], suggesting a causal link between preservation of mitochondrial integrity in the *cd47* null condition and the observed elevated hippurate levels. Interestingly, benzoate can be degraded by microbial processing to catechol compounds [41]. We found that catechol sulfate increased in *cd47* null mice when compared to WT but not basally in CD47 deficient T cells [13] (Supplemental Figure S2), suggesting a differential processing of benzoate mediated by differences in microbial populations between both genotypes. The levels of catechol sulfate were reduced in both groups as a consequence of ionizing radiation but remained higher in the irradiated *cd47* null mice. Benzoate processing is known to support carbohydrate metabolism [21], so it is possible that the differential processing observed can provide an advantage in radioprotection by supporting energy metabolism.

Whole body or thoracic lung radiation exposure can lead to radiation-induced lung disease which includes the development of pneumonitis and pulmonary fibrosis [14,42]. Therefore, accidental or therapeutic radiation exposure has consequences that impact quality of life. Moreover, an objective of radiation biodosimetry is to identify initial biomarkers that can reveal degrees of exposure to radiation for the successful coordination and clinical management of populations affected by an unexpected radiation exposure event [1]. Focusing on the lung as a sensitive tissue to radiation damage with both short-term and long-term consequences, our metabolomics approach identifies key metabolic pathways that are affected by radiation in both of our genotypes. Since we have observed increased survival in mice exposed to whole body irradiation and preservation of lung tissue viability with CD47 blockade, the findings in this study suggest radiation resistant *cd47* null lung tissue exhibits a unique biochemical signature associated with radioprotection that includes the regulation of redox handling, fatty acid synthesis for energy production, nucleotide metabolism and differential nutrient processing that may be mediated by enzymatic reactions regulated by TSP1/CD47 signaling or differential microbial processing. Therefore, targeting CD47 could be considered as a potential intervention to limit sequelae due to radiation-lung disease. Also, the markers associated with this radioprotection could be considered as potential intervention to mitigate the harmful effects of ionizing radiation exposure. 

## 4. Materials and Methods

### 4.1. Mouse Irradiation

WT and *cd47* null mice extensively back-crossed onto a C57Bl/6 background were obtained from Jackson Laboratories. All mice were bred in the same vivarium before use to ensure consistent housing conditions. Care and handling of animals occurred in an AAALAC approved facility in strict accordance with the recommendations in the Guide for the Care and Use of Laboratory Animals of the National Institutes of Health under protocol LP-012 approved by the Animal Care and Use Committee of the National Cancer Institute. Groups of 12-week old sex matched WT and *cd47* null mice (7–8 per group) received 7.6 Gy total body irradiation delivered by a Therapax DXT300 X-ray irradiator (Pantak, Inc., East Haven, CT 06512, USA) using 2.0-mm Al filtration (300 kVp) at a dose rate of 2.53 Gy/min. At 24 h post irradiation, lung tissue was harvested and flash frozen for metabolomic analysis [13].

### 4.2. Metabolite Analysis

Metabolomic profiling analysis was performed by Metabolon as previously described [13]. Each sample was accessioned into the Metabolon LIMS system and was assigned by the Laboratory Information Management System (LIMS) a unique identifier that was associated with the original source identifier only. This identifier was used to track all sample handling, tasks and results. The samples (and all derived aliquots) were tracked by the LIMS system. All portions of any sample were automatically assigned their own unique identifiers by the LIMS when a new task was created; the relationship of these samples was also tracked. All samples were maintained at −80 °C until processed. Samples were prepared using the automated MicroLab STAR^®^ system from Hamilton Company. A recovery standard was added prior to the first step in the extraction process for QC purposes. Sample preparation was conducted using aqueous methanol extraction process to remove the protein fraction while allowing maximum recovery of small molecules. The resulting extract was divided into four fractions: one for analysis by UPLC/MS/MS (positive mode), one for UPLC/MS/MS (negative mode), one for GC/MS, and one for backup. Samples were placed briefly on a TurboVap^®^ (Zymark, Portland, OR 97230, USA) to remove the organic solvent. Each sample was then frozen and dried under vacuum. Samples were then prepared for the appropriate instrument, either UPLC/MS/MS or GC/MS.

### 4.3. Liquid Chromatography/Mass Spectrometry (LC/MS, LC/MS^2^)

The LC/MS portion of the platform was based on a Waters ACQUITY UPLC and a Thermo-Finnigan LTQ mass spectrometer, which consisted of an electrospray ionization (ESI) source and linear ion-trap (LIT) mass analyzer. The sample extract was split into two aliquots, dried, then reconstituted in acidic or basic LC-compatible solvents, each of which contained 11 or more injection standards at fixed concentrations. One aliquot was analyzed using acidic positive ion optimized conditions and the other using basic negative ion optimized conditions in two independent injections using separate dedicated columns. Extracts reconstituted in acidic conditions were gradient eluted using water and methanol both containing 0.1% formic acid, while the basic extracts, which also used water and methanol, contained 6.5 mM ammonium bicarbonate. The MS analysis alternated between MS and data-dependent MS^2^ scans using dynamic exclusion.

### 4.4. Gas Chromatography/Mass Spectrometry (GC/MS)

The samples destined for GC/MS analysis were re-dried under vacuum desiccation for a minimum of 24 h prior to being derivatized under dried nitrogen using bistrimethyl-silyl-triflouroacetamide (BSTFA). The GC column was 5% phenyl and the temperature ramp was from 40 °C to 300 °C in a 16 min period. Samples were analyzed on a Thermo-Finnigan Trace DSQ fast-scanning single-quadrupole mass spectrometer using electron impact ionization. The instrument was tuned and calibrated for mass resolution and mass accuracy on a daily basis. The information output from the raw data files was automatically extracted as discussed below.

### 4.5. Bioinformatics

The informatics system consisted of four major components, the LIMS, data extraction and peak-identification software, data processing tools for QC and compound identification, and a collection of information interpretation and visualization tools for use by data analysts. The hardware and software foundations for these informatics components were the LAN backbone and a database server running Oracle 10.2.0.1 Enterprise Edition.

### 4.6. Statistical Calculation

Two types of statistical analysis were usually performed: (1) significance tests and (2) classification analysis. (1) For pair-wise comparisons we typically performed Welch’s t-tests and/or Wilcoxon’s rank sum tests. For other statistical designs, we performed appropriate ANOVA procedures (e.g., repeated measures ANOVA). (2) For classification we mainly use random forest analyses. Random forest analysis gives an estimate of how well we can classify individuals in a new data set into each group, in contrast to a t-test, which tests whether the unknown means for two populations are different. Random forest analysis creates a set of classification trees based on continual sampling of the experimental units and compounds. Then, each observation was classified based on the majority votes from all the classification trees. Statistical analyses were performed with the program “R” http://cran.r-project.org/.

## Figures and Tables

**Table 1:** Levels and ratios of reduced and oxidized glutathione.

Glutathione, Reduced (GSH)		Glutathione, Oxidized (GSSG)		GSH:GSSG	
Treatment	Average SEM	Treatment	Average SEM	Treatment	Ratio
WT	2.138 ± 0.6102	WT	1.007 ± 0.06007	WT	2.12
*cd47-/-*	2.830 ± 1.666	*cd47-/-*	0.9818 ± 0.05081	*cd47-/-*	2.88
WT + IR	0.9412 ± 0.1702	WT + IR	1.009 ± 0.03887	WT + IR	0.93
*cd47-/-* + IR	3.025 ± 2.002	*cd47-/-* + IR	0.9909 ± 0.01972	*cd47-/-* + IR	3.05

**Table 2:** Lysolipid metabolites fold change.

Lysolipid Metabolites	ANOVA Contrasts			
	Fold of Change			
	IR CTRL		CD47-/- WT	
	WT	CD47-/-	CTRL	IR
1-myristoylglycerophosphocholine (14:0)	4.01	1.35	1.23	0.41
2-myristoylglycerophosphocholine	3.99	1.09	1.29	0.35
1-palmitoylglycerophosphocholine (16:0)	3.68	1.08	1.38	0.41
2-palmitoylglycerophosphocholine	5.29	1.12	1.57	0.33
1-palmitoleoylglycerophosphocholine (16:1)	3.86	1.18	1.21	0.37
2-palmitoleoylglycerophosphocholine	4.29	1.17	1.30	0.35
1-stearoylglycerophosphocholine (18:0)	5.35	1.21	1.85	0.42
2-stearoylglycerophosphocholine	4.65	1.61	1.25	0.43
1-oleoylglycerophosphocholine (18:1)	4.08	1.02	1.52	0.38
2-oleoylglycerophosphocholine	4.66	1.10	1.31	0.31
1-linoleoylglycerophosphocholine (18:2n6)	4.19	1.13	1.43	0.39
2-linoleoylglycerophosphocholine	4.05	1.11	1.39	0.38
1-eicosatrienoylglycerophosphocholine (20:3)	2.28	0.94	1.10	0.45
1-arachidonoylglycerophosphocholine (20:4n6)	6.11	0.93	1.59	0.24
2-arachidonoylglycerophosphocholine	4.22	1.40	1.21	0.40
1-docosapentaenoylglycerophosphocholine (22:5n3)	3.31	1.57	0.84	0.40
2-docosapentaenoylglycerophosphocholine (22:5n3)	4.94	1.27	1.28	0.33
1-docosahexaenoylglycerophosphocholine (22:6n3)	3.96	1.28	1.29	0.42
2-docosahexaenoylglycerophosphocholine	5.23	1.34	1.45	0.37
1-palmitoylplasmenylethanolamine	1.57	1.21	0.84	0.64
1-stearoylplasmenylethanolamine	0.94	1.29	0.80	1.10
1-oleoylplasmenylethanolamine	1.60	1.16	0.95	0.69
1-palmitoylglycerophosphoethanolamine	1.34	1.14	0.82	0.70
2-palmitoylglycerophosphoethanolamine	4.49	1.05	1.63	0.38
1-stearoylglycerophosphoethanolamine	1.80	1.15	0.97	0.62
1-oleoylglycerophosphoethanolamine	1.33	0.93	0.97	0.68
2-oleoylglycerophosphoethanolamine	1.40	1.08	1.01	0.78
1-linoleoylglycerophosphoethanolamine	1.22	0.91	1.05	0.78
2-linoleoylglycerophosphoethanolamine	3.05	1.01	1.39	0.46
1-arachidonoylglycerophosphoethanolamine	1.22	1.04	1.07	0.91
2-arachidonoylglycerophosphoethanolamine	3.22	1.09	1.33	0.45
2-docosapentaenoylglycerophosphoethanolamine	7.07	1.09	1.79	0.28
2-docosahexaenoylglycerophosphoethanolamine*	4.08	1.11	1.60	0.43
1-eicosatrienoylglycerophosphoethanolamine	1.35	1.04	0.97	0.75
2-eicosapentaenoylglycerophosphoethanolamine	4.87	1.04	1.50	0.32
1-docosahexaenoylglycerophosphoethanolamine	1.29	1.18	1.02	0.93
1-eicosenoylglycerophosphoethanolamine (20:1n9)	1.42	0.97	1.09	0.74
1-palmitoylglycerophosphoinositol	1.05	0.98	0.92	0.87
1-stearoylglycerophosphoinositol	1.20	1.15	0.88	0.84
2-stearoylglycerophosphoinositol	1.55	1.04	1.11	0.75
1-oleoylglycerophosphoinositol	0.94	0.82	1.03	0.89
1-arachidonoylglycerophosphoinositol	0.96	0.96	0.87	0.86
2-arachidonoylglycerophosphoinositol	1.04	1.02	0.90	0.88
1-stearoylglycerophosphoserine	1.16	1.14	0.83	0.82
1-oleoylglycerophosphoserine	1.10	0.93	0.90	0.76
2-oleoylglycerophosphoserine	1.18	1.24	0.70	0.74
1-palmitoylglycerophosphoglycerol	1.08	1.26	0.70	0.82
2-palmitoylglycerophosphoglycerol	1.12	1.17	0.91	0.95
1-stearoylglycerophosphoglycerol	1.15	1.48	0.66	0.85
2-stearoylglycerophosphoglycerol	1.07	1.56	0.64	0.93
1-oleoylglycerophosphoglycerol	1.14	1.14	0.83	0.83
2-oleoylglycerophosphoglycerol	1.38	1.49	0.70	0.76

Red = *p* ≤ 0.05, fold change ≥ 1.00; Green = *p* ≤ 0.05, fold change < 1.00.

**Table 3:** Regulation of nucleotide metabolism intermediates.

		IR CTRL		CD47-/- WT	
	Metabolite	WT	CD47 -/-	CTRL	IR
Purine Metabolism, (Hypo)Xanthine/Inosine containing	inosine 5′-monophosphate (IMP)	0.11	0.08	2.80	2.09
	inosine	0.72	0.84	1.00	1.17
	hypoxanthine	0.99	1.07	0.91	0.98
	xanthine	1.07	1.12	0.92	0.96
	xanthosine	1.20	1.12	1.07	1.00
	urate	1.11	1.26	0.86	0.97
	allantoin	0.82	0.79	1.08	1.05
Purine Metabolism, Adenine containing	adenosine 5′-diphosphate (ADP)	1.05	0.89	1.20	1.02
	adenosine 5′-monophosphate (AMP)	0.69	0.46	1.63	1.09
	adenosine 3′-monophosphate (3′-AMP)	0.45	0.50	1.00	1.12
	adenosine 2′-monophosphate (2′-AMP)	1.19	1.46	0.72	0.89
	adenosine 3′,5′-cyclic monophosphate (cAMP)	0.91	1.10	0.93	1.13
	adenosine 3′,5′-diphosphate	0.99	1.24	0.88	1.11
	adenosine	1.10	0.95	1.25	1.07
	adenine	0.89	1.13	0.75	0.95
	N1-methyladenosine	1.02	0.96	1.04	0.98
Purine Metabolism, Guanine containing	guanosine 5′- monophosphate (5′-GMP)	0.32	0.15	3.01	1.37
	guanosine 3′-monophosphate (3′-GMP)	0.71	0.73	0.97	1.00
	guanosine	0.33	0.58	1.13	1.99
Pyrimidine Metabolism, Uracil containing	uridine monophosphate (5′ or 3′)	0.60	0.51	1.80	1.52
	uridine	1.12	1.15	1.03	1.05
	uracil	1.10	1.29	0.80	0.94
	pseudouridine	0.93	0.83	1.14	1.01
	3-ureidopropionate	1.18	1.08	1.03	0.94
	beta-alanine	0.91	0.90	1.13	1.12
Pyrimidine Metabolism, Cytidine containing	cytidine 5′-monophosphate (5′-CMP)	0.88	0.99	0.84	0.94
	cytidine-3′-monophosphate (3′-CMP)	0.18	0.22	0.94	1.15
	cytidine	1.08	1.07	0.90	0.89
	2′-deoxycytidine	2.11	1.35	1.11	0.71
Pyrimidine Metabolism, Thymine containing	thymidine	1.04	1.53	1.98	2.92
	thymine	1.37	1.48	1.52	1.64
Purine and Pyrimidine Metabolism	methylphosphate	0.87	1.15	0.76	0.99

Red = *p* ≤ 0.05, fold change ≥ 1.00; Green = *p* ≤ 0.05, fold change < 1.00.

## Supplementary Materials

The following are available online at https://www.mdpi.com/2218-1989/9/10/218/s1, Figure S1: Random forest analysis of WT and *cd47* null mice, Figure S2: Metabolites regulated in WT and *cd47* deficient T cells after ionizing radiation, Figure S3: Supporting metabolites of antioxidant and lipid metabolism.
