# Supplementary material for: Metabolomic Analysis Reveals Unique Biochemical Signatures Associated with Protection from Radiation Induced Lung Injury by Lack of cd47 Receptor Gene Expression
Source: Metabolites. 2019 Oct 8;9(10):218. doi: 10.3390/metabo9100218 (PMC6835245; doi:10.3390/metabo9100218)

Supplemental figure 1

A

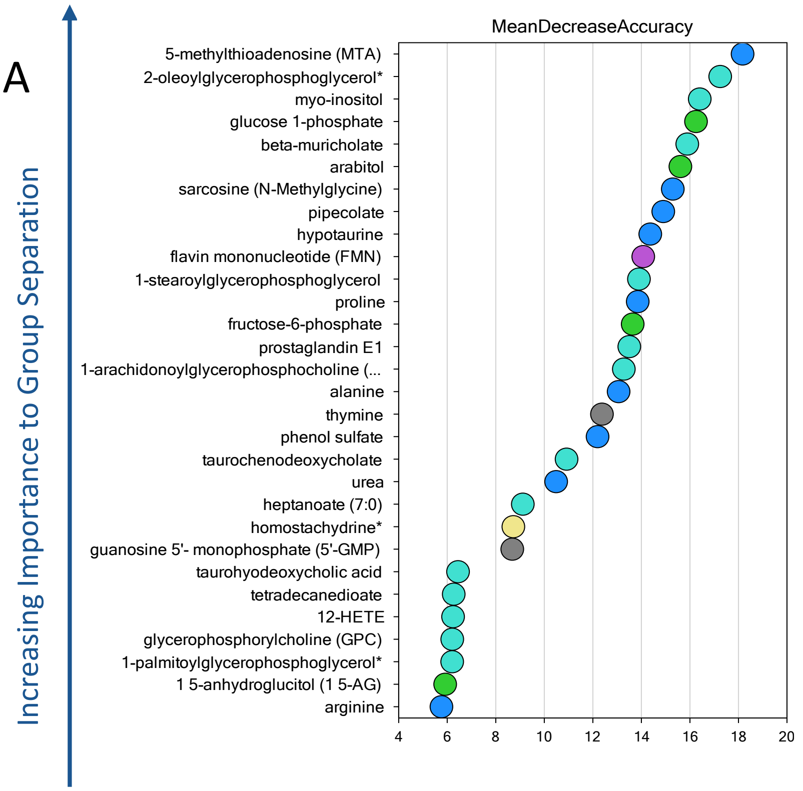

B

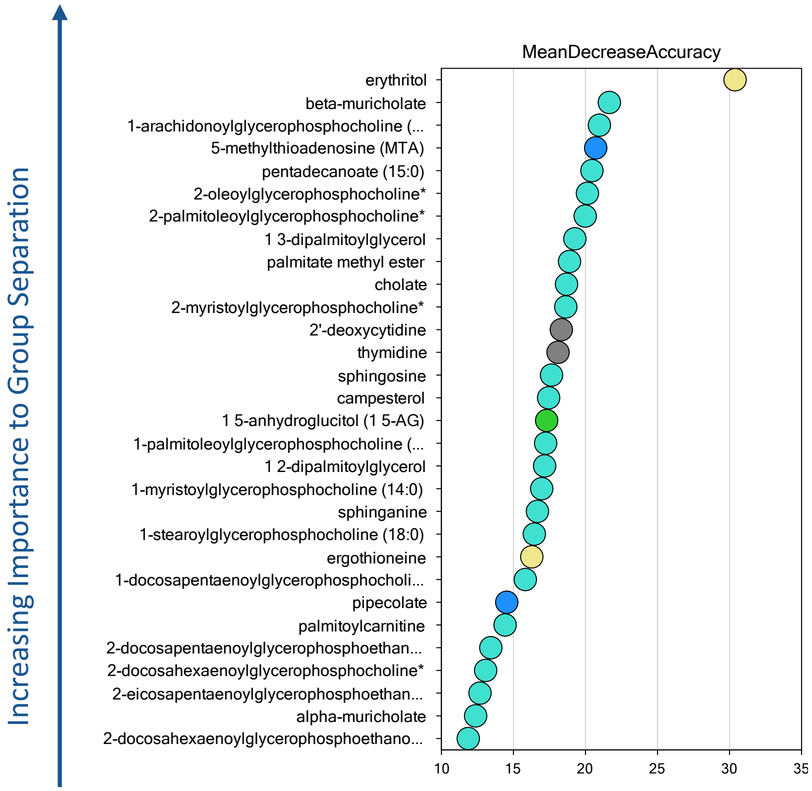

Supplemental figure 2

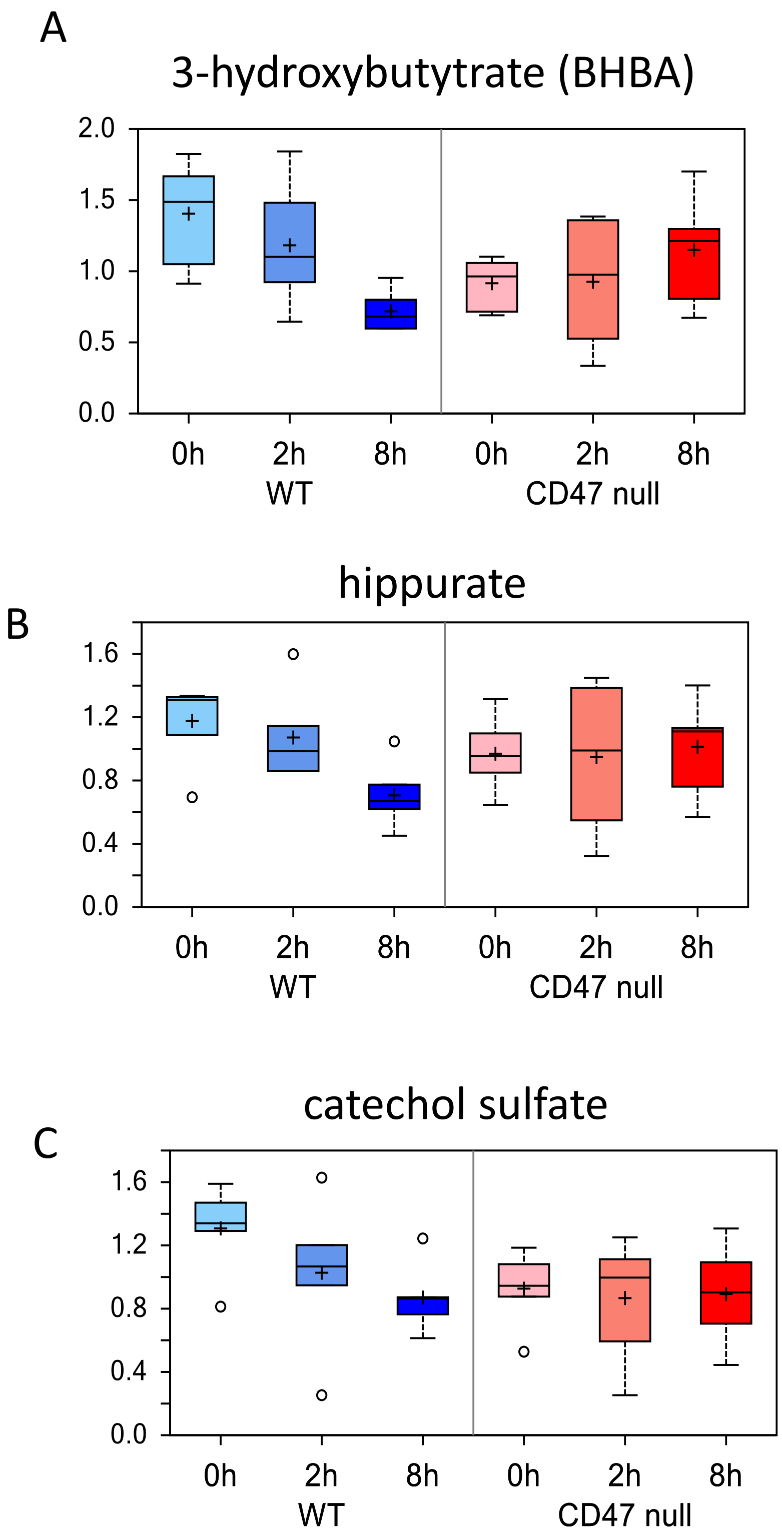

Supplemental figure 3

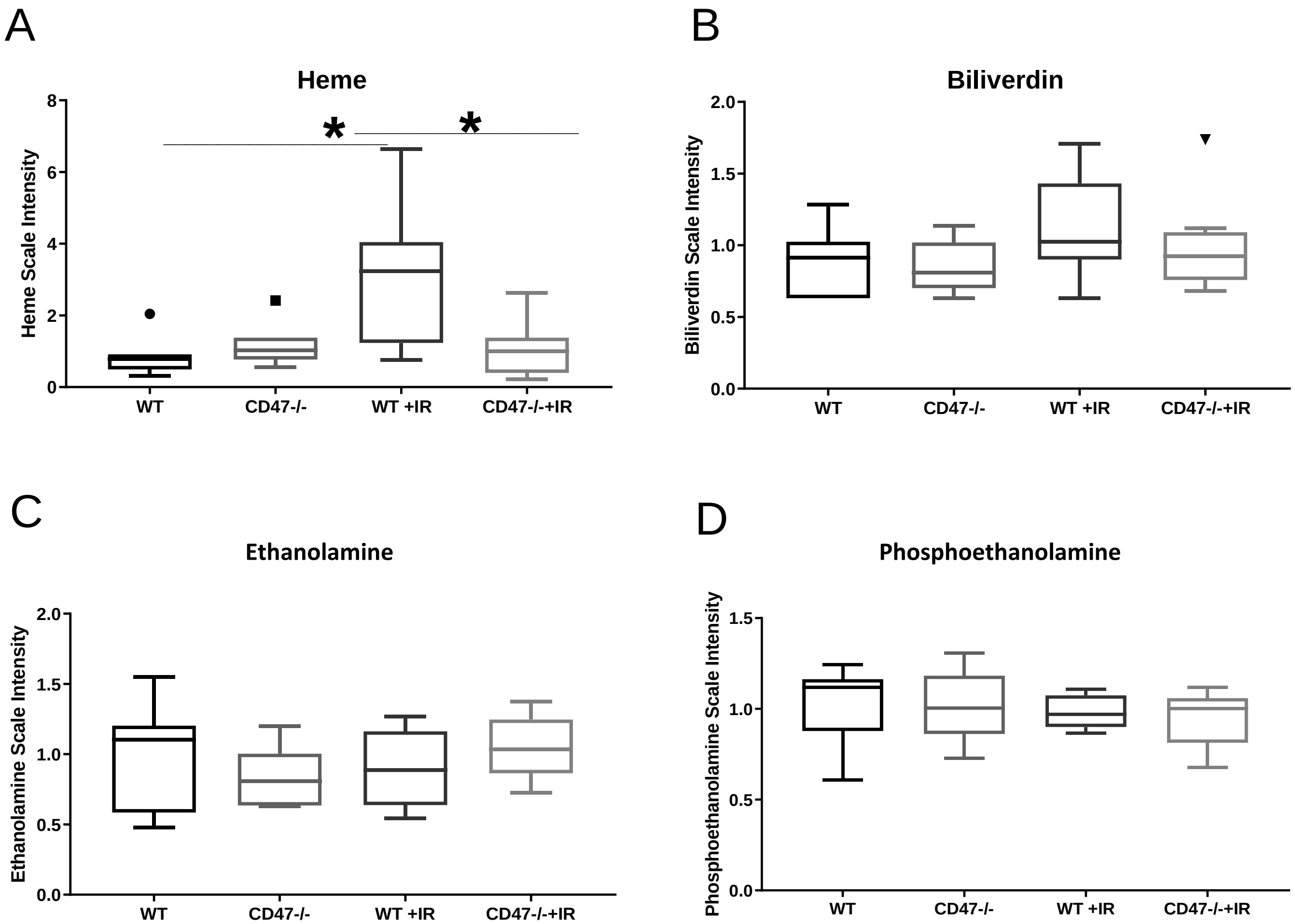

Supplement: Supplementary file 1 [file metabolites-09-00218-s001.pdf]
